# Effect of an Antacid (Aluminum Hydroxide/Magnesium Hydroxide/Simethicone) or a Proton Pump Inhibitor (Omeprazole) on the Pharmacokinetics of Tebipenem Pivoxil Hydrobromide (TBP-PI-HBr) in Healthy Adult Subjects

**DOI:** 10.1128/aac.01495-22

**Published:** 2023-03-21

**Authors:** Gina Patel, Vipul K. Gupta, Leanne Gasink, Floni Bajraktari, Yang Lei, Akash Jain, Praveen Srivastava, Angela K. Talley

**Affiliations:** a Patel Kwan Consultancy, LLC, Madison, Wisconsin, USA; b Spero Therapeutics, Cambridge, Massachusetts, USA

**Keywords:** aluminum hydroxide/magnesium hydroxide/simethicone, drug interaction, omeprazole, pharmacokinetics, tebipenem

## Abstract

Tebipenem pivoxil hydrobromide (TBP-PI-HBr) is a novel oral carbapenem prodrug being developed for the treatment of serious bacterial infections. This open-label, 3-period, fixed sequence study evaluated the effect of gastric acid-reducing agents, aluminum hydroxide/magnesium hydroxide/simethicone, and omeprazole on the pharmacokinetics (PK) of tebipenem (TBP), the active moiety, following coadministration with immediate release TBP-PI-HBr during fasting. In Period 1, subjects received a single oral dose of TBP-PI-HBr 600 mg (2 × 300 mg tablets). In Period 2, subjects received a single oral dose of aluminum hydroxide 800 mg/magnesium hydroxide 800 mg/simethicone 80 mg suspension co-administered with a single dose of TBP-PI-HBr 600 mg. In Period 3, subjects received a single oral dose of omeprazole 40 mg once daily over 5 days, followed by single dose administration of TBP-PI-HBr 600 mg on day 5. In each period, whole blood samples were obtained prior to, and up to 24 h, following TBP-PI-HBr dose administration in order to characterize TBP PK. A 7-day washout was required between periods. Twenty subjects were enrolled and completed the study. Following co-administration of TBP-PI-HBr with either aluminum hydroxide/magnesium hydroxide/simethicone or omeprazole, total TBP exposure (area under the curve [AUC]) was approximately 11% (geometric mean ratio 89.2, 90% confidence interval: 83,2, 95.7) lower, and Cmax was 22% (geometric mean ratio 78.4, 90% confidence interval: 67.9, 90.6) and 43% (geometric mean ratio 56.9, 90% confidence interval: 49.2, 65.8) lower, respectively, compared to administration of TBP-PI-HBr alone. Mean TBP elimination half-life (t_1/2_) was generally comparable across treatments (range: 1.0 to 1.5 h). Concomitant administration of TBP-PI-HBr with omeprazole or aluminum hydroxide/magnesium hydroxide/simethicone is not expected to impact the efficacy of TBP-PI-HBr, as there is minimal impact on TBP plasma AUC, which is the pharmacodynamic driver of efficacy. Co-administration was generally safe and well tolerated.

## INTRODUCTION

Tebipenem pivoxil hydrobromide (TBP-PI-HBr) is a novel prodrug of tebipenem (TBP), a carbapenem that undergoes rapid conversion to the active moiety TBP after oral administration. TBP exhibits broad spectrum *in vitro* activity against Gram-positive and Gram-negative pathogens, including extended-spectrum-β-lactamase (ESBL)-producing Enterobacterales, as well as fluoroquinolone-resistant strains (1). In animal models of infection, including the murine neutropenic thigh infection model and the murine ascending urinary tract infection (UTI) model, TBP demonstrated potent efficacy against ESBL-producing Escherichia coli (2, 3). In a hollow fiber infection model, every 8 h dosing with TBP produced rapid bacterial killing with minimal selection of resistant subpopulations (2). An unmet need exists for new oral antimicrobials to treat serious bacterial infections, especially due to resistant pathogens. TBP-PI-HBr is being developed for the treatment of complicated urinary tract infections and other serious bacterial infections, including those caused by multi-drug resistant Gram-negative pathogens.

In a Phase 1 single- and multiple-ascending dose study of TBP-PI-HBr, plasma concentrations of TBP increased in a linear and dose proportional manner, and the half-life of approximately 1 h was constant across a range of doses from 100 to 900 mg (4). Plasma concentrations of TBP after a 600 mg dose were similar under fed and fasted conditions. After multiple doses of TBP-PI-HBr 300 mg or 600 mg every 8 h for 14 days, TBP pharmacokinetic (PK) parameters were unchanged, and no accumulation was observed. In other Phase 1 studies, TBP-PI-HBr had no clinically significant effect on the QTc interval (5), and dosage adjustment is likely not required among subjects with mild or moderate renal impairment (6). TBP-PI-HBr was non-inferior to intravenous ertapenem in a phase 3 study of patients with complicated urinary tract infection or acute pyelonephritis for clinical and microbiological response (7).

A systematic review of potential drug-drug interactions with gastric acid-reducing medications found potentially clinically meaningful interactions only with some oral cephalosporins (8). Thus, concomitant administration of TBP-PI-HBr with a short-acting antacid combination (aluminum hydroxide/magnesium hydroxide/simethicone) and a proton pump inhibitor (PPI) (omeprazole) increases gastric pH, and may have an effect on the PK of TBP. The objectives of this study were to evaluate the effect of co-administration of a single dose of TBP-PI-HBr with a single dose of aluminum hydroxide/magnesium hydroxide/simethicone; an antacid or multiple doses of omeprazole; a proton pump inhibitor on the PK of TBP in healthy subjects.

## RESULTS

Twenty subjects were enrolled and completed the study, and all were included in PK and safety analyses. Baseline characteristics were compatible with a population of healthy subjects (Table 1).

**TABLE 1:** Baseline characteristics

Characteristic	Subjects (*n* = 20)
Age, yrs^*a*^	39.9 ± 8.7
Age range, yrs	18 to 53
Female, n (%)	9 (45)
Body mass index, kg/m^2^^*a*^	26.5 ± 2.9
Race, n (%)	
White	17 (85)
Black or African American	2 (10)
Asian	1 (5)
Ethnicity, n (%)	
Hispanic or Latino	17 (85)

a Mean ± standard deviation.

### Pharmacokinetics.

**FIG 1:**
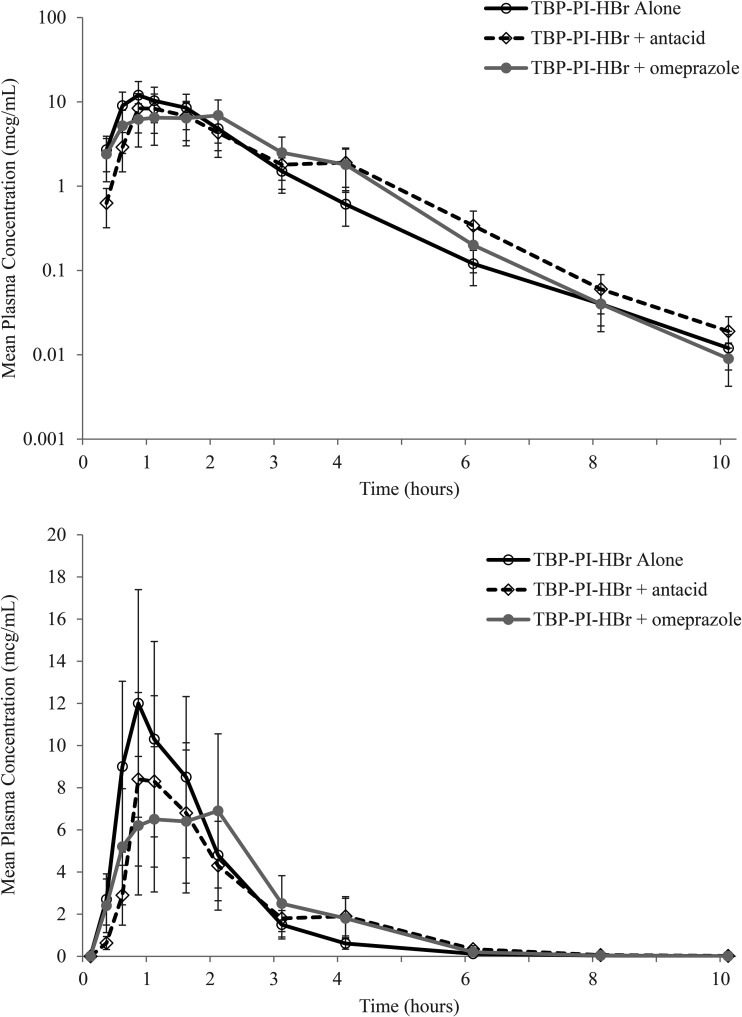
TBP mean (standard deviation) plasma concentration-time profile following a 600 mg dose of TBP-PI-HBr alone (Treatment A) and combined with aluminum hydroxide/magnesium hydroxide/simethicone (Treatment B) or omeprazole (Treatment C) in healthy subjects (linear top, semi-log bottom).

Plasma TBP concentrations were quantifiable by the first post dose sample time (0.25 h) for all subjects following TBP-PI-HBr alone (Treatment A) or in combination with omeprazole once daily (QD) (Treatment C), and for 12 out of 20 profiles following TBP-PI-HBr combined with aluminum hydroxide/magnesium hydroxide/simethicone (Treatment B). Mean peak TBP concentrations (Cmax) were lower (7.4, 10.1 μg/mL) for combination treatments relative to TBP-PI-HBr administered alone (12.9 μg/mL), occurring at 0.75 h post dose following TBP-PI-HBr alone or in combination with aluminum hydroxide/magnesium hydroxide/simethicone, and at 1 h post dose following TBP-PI-HBr combined with omeprazole QD, respectively (Fig. 1).

The median and range of individual T_max_ values were slightly delayed for TBP-PI-HBr in combination (1.0 h) with aluminum hydroxide/magnesium hydroxide/simethicone or omeprazole QD relative to TBP-PI-HBr administered alone (0.8 h) (Table 2).

**TABLE 2:** Pharmacokinetic parameters for each treatment group

Parameter	TBP-PI-HBr alone (Treatment A^*a*^) (*n* = 20)	TBP-PI-HBr + aluminum hydroxide/magnesium hydroxide/simethicone (Treatment B^*b*^) (*n* = 20)	TBP-PI-HBr + omeprazole QD (Treatment C^*c*^) (*n* = 20)
C_max_^*d*^ (μg/mL)	12.93 (41.7)	10.14 (32.1)	7.36 (48.3)
T_max_^*e*^ (h)	0.76 (0.50, 2.00)	1.00 (0.75, 4.00)	1.00 (0.75, 4.00)
t_1/2_^*f*^ (h)	1.47 ± 0.71	1.29 ± 0.42	0.99 ± 0.24
AUC^*d*^_0-t_ (μg*h/mL)	18.84 (30.2)	16.80 (23.3)	16.85 (29.2)
AUC^*d*^_0-inf_ (μg*h/mL)	18.87 (30.2)	16.83 (23.3)	16.87 (29.2)

a Treatment A: 600 mg (2 × 300 mg tablets) TBP-PI-HBr administered at Hour 0 on Day 1.

b Treatment B: 20 mL Mylanta Maximum Strength Classic Flavor (800 mg aluminum hydroxide/800 mg magnesium hydroxide/80 mg simethicone per 10 mL) oral suspension with 600 mg (2 × 300 mg tablets) TBP-PI-HBr at Hour 0 on Day 1.

c Treatment C: 40 mg (1 × 40 mg capsule) omeprazole administered QD at Hour -2 on Days 1 through 5, with 600 mg (2 × 300 mg tablets) TBP-PI-HBr administered at Hour 0 on Day 5.

d AUCs and Cmax are presented as geometric mean (geometric CV%).

e T_max_ values are presented as median (minimum, maximum).

f t_½_ values are presented as arithmetic mean ± SD.

Based on the statistical comparisons of natural log (ln)-transformed TBP AUC_0-t_ and AUC_0-inf_, administration of TBP-PI-HBr in combination with aluminum hydroxide/magnesium hydroxide/simethicone had no meaningful effect on TBP AUC, as the 90% CIs of the geometric mean ratios for the AUCs were within the standard equivalence limits of 80% to 125% (Table 3). The geometric mean ratios for AUCs indicated TBP exposure was approximately 11% lower for TBP-PI-HBr in combination with aluminum hydroxide/magnesium hydroxide/simethicone compared to TBP-PI-HBr alone. TBP Cmax with co-administration of aluminum hydroxide/magnesium hydroxide/simethicone and TBP-PI-HBr fell below the lower bound of the 90% CI (80% to 125% limits) of the geometric mean ratio for Cmax (67.9%). Co-administration of aluminum hydroxide/magnesium hydroxide/simethicone with TBP-PI-HBr decreased TBP Cmax by approximately 22%.

**TABLE 3:** Statistical comparisons of plasma PK parameters for TBP + aluminum hydroxide/magnesium hydroxide/simethicone versus TBP alone and TBP + omeprazole versus TBP alone (PK population)

	TBP-PI-HBr + aluminum hydroxide/magnesium hydroxide/simethicone (Treatment B)^*a*^	TBP-PI-HBr alone (Treatment A)^*b*^			
Parameter^*c*^	Geometric LSMs^*d*^	Geometric LSMs	GMR^*e*^ (%)	90% confidence interval	Intra-subject CV%^*f*^
AUC_0-t_ (μg*h/mL)	16.8	18.8	89.2	83.1 - 95.7	13.2
AUC_0-inf_ (μg*h/mL)	16.8	18.9	89.2	83.2 - 95.7	13.2
Cmax (μg/mL)	10.1	12.9	78.4	67.9 - 90.6	27.6
	TBP-PI-HBr + Omeprazole QD (Treatment C^*g*^)	TBP-PI-HBr alone (Treatment A)			
Parameter	Geometric LSMs^*d*^	Geometric LSMs	GMR^*e*^ (%)	90% confidence interval	Intra-subject CV%^*f*^
AUC_0-t_ (μg*hr/mL)	16.9	18.8	89.4	83.4 - 95.9	13.2
AUC_0-inf_ (μg*hr/mL)	16.9	18.9	89.4	83.4 - 95.9	13.2
Cmax (μg/mL)	7.4	12.9	56.9	49.3 - 65.8	27.6

a Treatment B: 20 mL maximum strength classic flavor (800 mg aluminum hydroxide/800 mg magnesium hydroxide/80 mg simethicone per 10 mL) oral suspension with 600 mg (2 × 300 mg tablets) TBP-PI-HBr at Hour 0 on Day 1.

b Treatment A: 600 mg (2 × 300 mg tablets) TBP-PI-HBr administered at hour 0 on day 1.

c Parameters were ln-transformed prior to analysis.

d Geometric least-squares means (LSMs) were calculated by exponentiating the LSMs derived from the ANOVA.

e Geometric Mean Ratio (GMR) = 100 × (test/reference).

f Intra-subject CV% was calculated as 100 × square root(exp[MSE]-1), where MSE = Residual variance from ANOVA.

g Treatment C: 40 mg (1 × 40 mg capsule) omeprazole administered QD at Hour -2 on Days 1 through 5, with 600 mg (2 × 300 mg tablets) TBP-PI-HBr administered at Hour 0 on Day 5.

Similarly, based on the statistical comparisons of ln-transformed TBP AUC_0-t_ and AUC_0-inf_, administration of TBP-PI-HBr in combination with omeprazole QD had no meaningful effect on TBP plasma exposure, as the 90% CIs of the geometric mean ratios for the AUCs were within the bioequivalence limits of 80% to 125% (Table 3). The geometric mean ratios for AUCs indicated that TBP exposure was approximately 11% lower for TBP-PI-HBr in combination with omeprazole QD compared to TBP-PI-HBr alone. Administration of omeprazole QD with TBP-PI-HBr did have an effect on TBP Cmax, as the lower bound (49.3%) of the 90% CI of the geometric mean ratio for Cmax fell below the 80% to 125% limits. Coadministration of omeprazole QD with TBP-PI-HBr decreased TBP Cmax by approximately 43%.

### Tolerability.

Overall, 12 (60%) subjects reported 24 treatment-emergent adverse events (TEAEs), where the most common were those of the gastrointestinal tract (Table 4). All TEAEs were mild in severity and resolved during the study period. No deaths, serious AEs, or discontinuations due to AEs were reported. No clinically significant electrocardiogram (ECG), vital sign, or clinical laboratory abnormalities were observed.

**TABLE 4:** Incidence of adverse events by treatment (safety population)^*a*^

	No. (%) of Subjects					
	Treatment					
Adverse events preferred term	A^*b*^	B^*c*^	C1^*d*^	C2^*e*^	C combined^*f*^	Overall
No. with TEAEs	3	9 (45)	2 (10)	3 (15)	5 (25)	12 (60)
Any individual TEAE						
Pinguecula	0	1 (5)	0	0	0	1 (5)
Abdominal discomfort	0	0	1 (5)	1 (5)	2 (10)	2 (10)
Constipation	0	2 (10)	0	0	0	2 (10)
Diarrhea	2 (10)	2 (10)	0	0	0	2 (10)
Flatulence	1 (5)	0	0	1 (5)	1 (5)	1 (5)
Vessel puncture site pain	0	2 (10)	0	0	0	2 (10)
Headache	0	1 (5)	1 (5)	0	1 (5)	2 (10)
Limited symptom panic attack	0	1 (5)	0	0	0	1 (5)
Cough	0	1 (5)	0	0	0	1 (5)
Dermatitis contact	0	1 (5)	0	0	0	1 (5)
Pruritus	1 (5)	0	0	1 (5)	1 (5)	2 (10)
Rash papular	1 (5)	0	0	0	0	1 (5)
Rash pruritic	0	0	0	1 (5)	1 (5)	1 (5)

a Adverse events are classified according to MedDRA Version 23.0. TEAE = Treatment-emergent adverse event. Although a subject may have had 2 or more adverse events, the subject was counted only once within a category. The same subject may appear in different categories.

b Treatment A: 600 mg (2 × 300 mg tablets) TBP-PI-HBr administered at Hour 0 on Day 1.

c Treatment B: 20 mL Mylanta Maximum Strength Classic Flavor (800 mg aluminum hydroxide/80 mg simethicone per 10 mL) oral suspension with 600 mg (2 × 300 mg tablets) TBP-PI-HBr at Hour 0 on Day 1.

d Treatment C: 40 mg (1 × 40 mg capsule) omeprazole administered QD at Hour -2 on Days 1 through 5, with 600 mg (2 × 300 mg tablets) TBP-PI-HBr administered at Hour 0 on Day 5.

e Treatment C1: Omeprazole alone (prior to coadministration of TBP-PI-HBr).

f Treatment C2: TBPM-PI-HBr + Omeprazole (after coadministration of TBP-PI-HBr).

## DISCUSSION

Aluminum hydroxide/magnesium hydroxide is an over-the-counter non-systemic antacid commonly used in the general population to relieve heartburn, acid indigestion, and sour stomach. The change in gastric pH is achieved by neutralizing secreted gastric acid rather than inhibiting the secretion of the acid (8). The aluminum hydroxide/magnesium hydroxide/simethicone product used in this study also contains simethicone, which is added to alleviate discomfort from bloating due to the accumulation of gases in the digestive system. An increase in gastric pH after administration of non-systemic antacids may directly affect the absorption and bioavailability of other drugs (8). Drug interactions with antacids also may be caused by changes in gastric emptying, intraluminal binding or chelation, and intraluminal pH or urinary pH (8, 9). Systematic reviews have reported that drug-drug interactions with antacids or gastric suppressing medications are common and often underrecognized (8, 10).

Omeprazole is a PPI that blocks the final step of acid production. This effect is dose-related and leads to inhibition of both basal and stimulated acid secretion, irrespective of the stimulus (8). Omeprazole is used for the treatment of duodenal and gastric ulcers, heartburn, esophagitis, and pathological hypersecretory conditions. After oral administration of 10 to 40 mg, the onset of the antisecretory effect of omeprazole occurs within 1 h, with the maximum effect occurring within 2 h. Inhibition of secretion is about 50% of maximum at 24 h, and the duration of inhibition lasts up to 72 h. The inhibitory effect of omeprazole on acid secretion increases with repeated, once daily dosing, and reaching a plateau after 4 days. Due to the profound and long-lasting inhibition of gastric acid secretion, omeprazole may interfere with absorption of drugs where gastric pH is an important determinant of bioavailability. In addition, omeprazole is an inhibitor of cytochrome (CYP) P450 2C19, and can be used as a sensitive substrate for studies investigating drug-drug interactions (11). While food has no effect on the bioavailability of TBP, and TBP has a low risk of drug-drug interactions, it was important to determine if coadministration of omeprazole had any effects on TBP exposure.

PPIs suppress gastric acid secretion to a greater extent for a longer duration than some other gastric pH-elevating agents, such as H2 blockers (e.g., famotidine) and antacids. As both antacids and PPIs are commonly used in clinical practice, the PPI omeprazole was selected for evaluation in this study, as any potential effect on TBP PK was likely to be maximized with the co-administration of a PPI. Aluminum hydroxide/magnesium hydroxide/simethicone is a short-acting acid-neutralizing agent, while PPIs inhibit the H+/K+ ATPase pump irreversibly with an effect that lasts approximately 7 to 10 days after discontinuation. As such, a fixed sequence design was selected where PPI dosing was conducted during the last dosing period of the study.

The washout period of 7 days between dosing periods was considered sufficient to prevent carryover effects of the preceding treatment, based on TBP t_½_ of approximately 1 h, and the duration of action of aluminum hydroxide/magnesium hydroxide/simethicone.

A previous study performed with a pediatric granule formulation of tebipenem (Orapenem) marketed in Japan demonstrated a reduction in TBP Cmax by 44% to 63% and AUC_0-inf_ by 21% to 33% when co-administered with either famotidine (a histamine-2 receptor antagonist) or an antacid (data on file). The TBP-PI-HBr formulation used in this study is a salt form developed for greater stability. Based on the effects of gastric acid reduction (increased gastric pH) on the PK of TBP, an assessment of the impact of concomitant administration of acid-neutralizing/reducing agents on the TBP PK profile following administration of TBP-PI-HBr was warranted.

Coadministration of either a single dose of aluminum hydroxide/magnesium hydroxide/simethicone or multiple doses of omeprazole with a single dose of TBP-PI-HBr had varying effects on Cmax and AUC. TBP AUC was approximately 11% lower for TBP-PI-HBr in combination with aluminum hydroxide/magnesium hydroxide/simethicone or omeprazole, as compared with TBP-PI-HBr alone. However, TBP plasma Cmax decreased by 22% and 43% when TBP-PI-HBr was co-administered with a single dose of aluminum hydroxide/magnesium hydroxide/simethicone or with omeprazole QD for 5 days, respectively. While geometric mean TBP Cmax were higher for TBP-PI-HBr alone, slight delay in TBP T_max_ and mean TBP concentrations in the early terminal phase were higher for combination treatments, suggesting an extended absorption phase relative to TBP-PI-HBr administered alone. The pharmacokinetic-pharmacodynamic (PK-PD) index that best described TBP efficacy was determined to be fAUC/MIC*1/τ based on neutropenic murine thigh infection model (2, 4). Since plasma AUC of TBP remained relatively unchanged in the presence of co-administered agents, the observed decrease in TBP Cmax is not considered to be clinically meaningful. Administration of a single oral dose of TBP-PI-HBr alone and co-administration with aluminum hydroxide/magnesium hydroxide/simethicone or with omeprazole appeared generally safe and well tolerated, and no new safety concerns were identified during the study.

In summary, the study results confirm no clinically meaningful drug-drug interaction with co-administration of TBP-PI-HBr and omeprazole or aluminum hydroxide/magnesium hydroxide/simethicone.

### Study highlights.

**(i) What is the current knowledge on the topic?** An increase in gastric pH after administration of non-systemic antacids may directly affect the absorption and bioavailability of other drugs. Drug interactions with antacids may also be caused by changes in gastric emptying, intraluminal binding or chelation, and intraluminal pH or urinary pH. Drug-drug interactions with antacids or gastric suppressing medications are common and often go underrecognized.

**(ii) What question did this study address?** The objectives of this study were to evaluate the effect of co-administration of a single dose of TBP-PI-HBr with a single dose of aluminum hydroxide/magnesium hydroxide/simethicone, an antacid, or multiple doses of omeprazole, a proton pump inhibitor, on the PK of TBP as well as safety and tolerability of TBP-PI-HBr.

**(iii) What does this study add to our knowledge?** These results indicate the absence of a clinically meaningful effect of antacids or an antisecretory drug on the PK of TBP, a novel oral carbapenem.

**(iv) How might this change clinical pharmacology or translational science?** If approved, oral TBP-PI-HBr may be administered for treating serious bacterial infections without concern that concomitant use of antacids or antisecretory drugs will alter efficacy of TBP.

## MATERIALS AND METHODS

The study was conducted in accordance with the U.S. Code of Federal Regulations and ethical principles of the Declaration of Helsinki, Good Clinical Practices, and the International Council for Harmonisation guidelines. The study protocol and all amendments were reviewed by an Institutional Review Board for the study center (clinicaltrials.gov: NCT04368585; Advarra, Columbia, MD). Written informed consent was obtained from each subject prior to participation in any study procedures.

### Study design.

This was an open-label, 3-period, fixed sequence, drug-drug interaction study. In Period 1, day 1, subjects received a single oral dose of TBP-PI-HBr 600 mg (2 × 300 mg tablets) at hour 0 (Treatment A). In Period 2, day 1, subjects received a single oral dose of 20 mL aluminum hydroxide 800 mg/magnesium hydroxide 800 mg/simethicone 80 mg suspension with a single oral dose of TBP-PI-HBr 600 mg at hour 0 (Treatment B). In Period 3, days 1 through 5, subjects received a single oral dose of omeprazole 40 mg once daily (QD), at hour -2 (relative to the expected dosing time of TBP-PI-HBr on day 5) (Treatment C). On day 5, a single oral dose of 600 mg TBP-PI-HBr was administered at hour 0. A washout period of at least 7 days was included between dosing in Period 1 and Period 2, and between dosing in Period 2 and Period 3.

On day -1 of Period 1, subjects reported to the clinic and commenced a 10-h overnight fast. On the morning of day 1 of Period 1, subjects received study drug with 240 mL of tap water. Water (except water provided with each dosing) was restricted 1 h prior to and 1 h after each study drug administration, but was allowed as needed at all other times. Subjects continued to fast for at least 4 h post dose on day 1 of Period 1. Subjects also fasted overnight for at least 10 h prior to dosing on day 1 of Period 2, and on day 5 of Period 3. Subjects continued the fast for at least 4 h post dose on day 1 of Period 2, and on day 5 of Period 3. On days 1 through 4 of Period 3, subjects fasted for at least 1 h prior to dosing, and remained fasted for at least 2 h post dose. In Periods 1 and 3, each dose of TBP-PI-HBr or omeprazole was administered orally with approximately 240 mL of water. In Period 2, 20 mL of aluminum hydroxide/magnesium hydroxide/simethicone suspension was administered orally, and the dosing vessel was rinsed twice with 60 mL of water to ensure complete consumption of the dose. TBP-PI-HBr was administered immediately after the aluminum hydroxide/magnesium hydroxide/simethicone dose with the remaining 100 mL of dosing water.

### Subject selection.

Adult men or women 18 to 55 years of age were eligible if they had a body mass index (BMI) ≥ 18.0 and ≤ 32.0 kg/m^2^, and were medically healthy without clinically significant findings on medical history, physical examination, vital signs, 12-lead ECG, or clinical laboratory testing (hematology, biochemistry, coagulation, and urinalysis). Women of childbearing potential were required to use an acceptable form of contraception from screening and throughout the study.

Subjects were excluded for a history of any clinically significant medical or psychiatric condition that could interfere with the conduct of the study. In addition, subjects were excluded for a history or presence of alcoholism or drug abuse with the past 2 years; history of significant allergic disease or hypersensitivity reactions to study drug or related compounds; known genetic metabolism anomaly associated with carnitine deficiency (e.g., carnitine transporter defect, methylmalonic aciduria, propionic acidemia); or any condition that could affect drug absorption. Subjects with positive urine drug or alcohol results at screening or the first study visit, or positive results for human immunodeficiency virus (HIV-1 and 2), hepatitis B surface antigen (HBsAg), or hepatitis C virus (HCV) at screening were excluded. Seated blood pressure < 90/40 mmHg or > 140/90 mmHg or heart rate <40 bpm or >99 bpm at the screening visit; QT interval corrected for heart rate using Fridericia’s formula (QTcF) interval >460 msec (males) or >470 msec (females) or abnormal clinically significant ECG findings at screening, were reasons for exclusion. Subjects had to have estimated creatinine clearance <80 mL/min at screening. Use of any prescription or non-prescription medications within 14 days; use of any drugs known to be significant inducers of cytochrome P450 (CYP) 2C19 or CYP3A4 enzymes and/or P-glycoprotein; or use of any gastric acid-reducing medications; valproic acid or divalproex sodium; probenecid; herbal products prior to the dosing, and throughout the study, was prohibited.

### Study assessments.

Study assessments included complete physical examinations, vital signs (systolic and diastolic blood pressure, heart rate, respiratory rate, and oral temperature), 12-lead ECG, clinical laboratory tests (e.g., hematology, biochemistry, coagulation, and urinalysis), and monitoring for adverse events (AEs). Whole blood samples were collected to determine TBP concentrations at the following time points: pre-dose (0) and 0.25, 0.5, 0.75, 1, 1.5, 2, 3, 4, 6, 8, 10, 12, 16, and 24 h post dose. Whole blood samples were assayed for TBP using a validated liquid chromatography tandem mass spectrometry (LC-MS/MS) method (Charles River Laboratories) (12). TBP blood concentrations were converted to plasma before the PK analysis using the following formula: Plasma concentration = reported blood concentration × 3.6, where 3.6 represents the product of 1/plasmatocrit value of 1.8 (using an average plasmatocrit value of 0.55) and isopropyl alcohol (IPA) dilution factor of 2. IPA was added as a stabilizer during blood sample collections to prevent conversion of TBP-PI-HBr to TBP post sample collection. Plasma concentrations less than the lower limit of quantitation (LLOQ, 7.2 ng/mL) were reported as 0, and treated as missing for PK analyses.

### Pharmacokinetic analysis.

The following PK parameters were calculated using noncompartmental methods Phoenix WinNonlin version 8.1 (Pharsight Corporation) based on plasma TBP concentrations: area under the concentration-time curve from time zero to the last observed non-zero concentration calculated by the linear up log down trapezoidal method (AUC_0-t_); area under the concentration-time curve from time zero extrapolated to infinity (AUC_0-inf_); percent of AUC_0-inf_ extrapolated, represented as (1 − AUC_0-t_/AUC_0-inf_)*100 (AUC_%extrap_); maximum observed concentration (Cmax); time to reach Cmax (T_max_); apparent first-order terminal elimination rate constant (Kel) calculated from a semi-log plot of the plasma concentration versus time curve; apparent first-order terminal elimination t_1/2_ calculated as 0.693/Kel; and time to reach C_last_ (T_last_).

### Statistical analysis.

The sample size of 20 subjects was determined based on the expectation that data from 18 evaluable (complete) subjects would provide adequate precision estimate for the geometric mean ratios, according to the within-subject variability of TBP parameters observed in previously conducted studies conducted during fasting with a 600 mg dose with an immediate release formulation (4, 6). As an illustration, an analysis of AUC, with an observed GMR of 0.7, would be estimated to have a 90% CI spanning from 0.59 to 0.83, assuming the analysis included 18 subjects and within-subject SD (log) was 0.3.

Analyses of variance (ANOVA) was performed on the ln-transformed plasma TBP PK parameters (AUC_0-t_, AUC_0-inf_, and Cmax) to assess the effect of aluminum hydroxide/magnesium hydroxide/simethicone and omeprazole on the PK of TBP. The ANOVA model included treatment as a fixed effect and subject as a random effect. The period and sequence effect could not be tested due to the fixed sequence study design. Each ANOVA included a calculation of least-squares means (LSM) as well as the difference between treatment LSM. Point estimates and 90% confidence intervals (CIs) were constructed for the relevant contrasts from the ANOVA models. The point estimates and 90% CIs were backtransformed to give estimates of the ratios of the geometric LSM and corresponding 90% CIs. Estimated geometric means were also presented for each treatment. Comparisons were TBP-PI-HBr plus aluminum hydroxide/magnesium hydroxide/simethicone (Treatment B) versus TBP-PI-HBr alone (Treatment A) and TBP-PI-HBr plus omeprazole (Treatment C) versus TBP-PI-HBr alone (Treatment A). All statistical analyses were conducted using SAS Version 9.4 (Cary, NC).
